# Draft Genome Sequence of the First Hypermucoviscous *Klebsiella quasipneumoniae* subsp. *quasipneumoniae* Isolate from a Bloodstream Infection

**DOI:** 10.1128/genomeA.00952-15

**Published:** 2015-09-17

**Authors:** Fabio Arena, Lucia Henrici De Angelis, Filippo Pieralli, Vincenzo Di Pilato, Tommaso Giani, Francesca Torricelli, Marco Maria D’Andrea, Gian Maria Rossolini

**Affiliations:** a Department of Medical Biotechnologies, University of Siena, Siena, Italy; b Internal Medicine Unit, Careggi University Hospital, Florence, Italy; c Department of Surgery and Translational Medicine, University of Florence, Florence, Italy; d Genetic Diagnostic Unit, Florence Careggi University Hospital, Florence, Italy; e Department of Experimental and Clinical Medicine, University of Florence, Florence, Italy; f Clinical Microbiology and Virology Unit, Florence Careggi University Hospital, Florence, Italy

## Abstract

*Klebsiella quasipneumoniae* is a recently described species, formerly identified as *K. pneumoniae* phylogroup KpII. Information on pathogenic and virulence potential of this species are lacking. We sequenced the genome of a hypermucoviscous *K. quasipneumoniae* clinical isolate showing a virulence genes content (*allABCDRS*, *kfuABC*, and *mrkABCDFHIJ*) peculiar to hypervirulent *K. pneumoniae* strains.

## GENOME ANNOUNCEMENT

*Klebsiella pneumoniae* is a major human pathogen causing hospital- and community-acquired infections (1, 2). The latter also include invasive infections characterized by pyogenic liver abscesses with possible dissemination to distant sites (3). The majority of strains causing these infections exhibit a “hypermucoviscous” phenotype which is considered a distinctive virulence factor (3, 4). Recent taxonomic studies have demonstrated that strains formerly classified in *K. pneumoniae* phylogroups KpII and KpIII actually belong to two new sister species, namely, *Klebsiella quasipneumoniae* and *Klebsiella variicola*, respectively (5, 6). The former species includes in turn two subspecies, *K. quasipneumoniae* subsp. *quasipneumoniae* and *K. quasipneumoniae* subsp. *similipneumoniae* (5).

Sequencing of the first genome of a *K. variicola* showing a hypermucoviscous phenotype has recently been announced (7).

In this report, we announce the first draft genome sequence of the *K. quasipneumoniae* subsp. *quasipneumoniae* strain (FI_HV_2014), characterized by a hypermucoviscous phenotype. The strain was isolated from a Peruvian patient hospitalized in Italy with biliary tract and bloodstream infection. To the best of our knowledge, this is the first reported hypermucoviscous strain of *K. quasipneumoniae* subsp. *quasipneumoniae.*

FI_HV_2014 genomic DNA was subjected to whole-genome sequencing with the MiSeq platform (Illumina Inc., San Diego, CA), using a 2×250-paired-end approach. In total 2,781,628 reads were obtained, with an average coverage of 84× and an estimated genome size of 5,335,587 bp. Reads were assembled using A5-miseq software (8) into 107 contigs and 84 scaffolds (*N*_50_ contig sizes of 316,178 bp). Scaffolds annotated using the NCBI Prokaryotic Genome Annotation Pipeline (release 2013) contained 4,799 coding sequences (CDS). The average GC content of the chromosome was about 55%. The species identification was deduced from the presence and nature of the chromosomal *bla*_OKP-type_ beta-lactamase, characteristic of *K. quasipneumoniae* subsp. *quasipneumoniae* (9), and confirmed by *fusA*, *gapA*, *gyrA*, *leuS*, and *rpoB* gene analysis (5). The predicted DNA-DNA hybridization (DDH), estimated using the GGDC 2.0 software (10), between FI_HV_2014 and type strains of *K. quasipneumoniae* subsp. *quasipneumoniae* (01A030, accession no. CCDF00000000), *K. quasipneumoniae* subsp. *similipneumoniae* (07A044, accession no. CBZR000000000), *K. variicola* (342, accession no. CP000964) and *K. pneumoniae* subsp. *pneumoniae* (DSM 30104, accession no. AJJI00000000) were 93.6%, 72.1%, 52.3%, and 53.7%, respectively.

A screening for (putative) virulence genes present in the BIGSdb-Kp database (http://bigsdb.web.pasteur.fr/perl/bigsdb/bigsdb.pl?db=pubmlst_klebsiella_seqdef_public&page=downloadAlleles), performed using the BLASTn tool, revealed (i) an *allABCDRS* operon (11), responsible for the allantoin anaerobic assimilation, linked with *arcC*, *fdrA*, *gcl*, *glxKR*, *ybbWY*, *ylbEF*, *KP1_1364*, *KP_1371*, and *hyi* genes in the same contig (accession no. AB115590); (ii) the *kfuABC* system (12), responsible for ferric iron uptake, and (iii) the mannose-resistant *Klebsiella*-like (type III) fimbriae cluster, *mrkABCDFHIJ* (13). Interestingly, the allantoin operon was not present in the genome of the *K. quasipneumoniae* subsp. *quasipneumoniae* type strain, suggesting recent horizontal acquisition by FI_HV_2014. The strain possessed a new capsular *wzi* allele (not present in the BIGSdb database). Notably, the *rmpA* and *rmpA2* genes, previously associated with the hypermucoviscous phenotype in *K. pneumoniae* strains (4), were not found in the genome of FI_HV_2014, suggesting the presence of a different capsular regulation mechanism.

### Nucleotide sequence accession numbers.

This whole-genome shotgun project has been deposited at DDBJ/EMBL/GenBank under the accession no. LGAL00000000. The version described in this paper is version LGAL01000000.
